# Diagnostic Value of Serum CFL1 and TAGLN2 for Non-Metastatic Gastric Cancer: A Retrospective and Prospective Real-World Study

**DOI:** 10.3390/cancers18101598

**Published:** 2026-05-14

**Authors:** Kewei Du, Shan Gao, Haichen Wang, Wenfei Hu, Caiyan Gao, Xiaoling Zheng, Xiaoqing Meng, Tuanjie Che, Shangdi Zhang

**Affiliations:** 1 Laboratory Medicine Center, The Second Hospital & Clinical Medical School, Lanzhou University, Lanzhou 730030, China; 2 Cuiying Biomedical Research Center, The Second Hospital & Clinical Medical School, Lanzhou University, Lanzhou 730030, China; 3 School of Mathematics and Statistics, Shanxi University, Taiyuan 030006, China; 4 Department of Clinical Nutrition, The Second Hospital & Clinical Medical School, Lanzhou University, Lanzhou 730030, China; 5 Innovation Center of Functional Genomics and Molecular Diagnostics Technology of Gansu Province, Lanzhou 730010, China; 6 Lanzhou Centers for Animal Disease Control & Prevention, Lanzhou 730030, China

**Keywords:** gastric cancer, early diagnosis, tumor biomarkers, proteomics

## Abstract

**Background**: Timely screening and diagnosis are critical for improving prognosis and survival in gastric cancer. Building on prior serum proteomics findings, this study evaluated two novel early-stage diagnostic biomarkers, CFL1 and TAGLN2, using retrospective and prospective cohorts. **Methods**: Retrospective analysis included 176 non-metastatic gastric cancer patients, 88 healthy controls, and patients with non-gastric non-metastatic cancers/malignancies (*n* = 143). A prospective study enrolled 88 gastroenterology patients with unknown cancer status before sampling; conventional markers, including CEA, AFP, CA199, and CA125, were measured for comparison. **Results**: Retrospectively, CFL1 and TAGLN2 achieved approximately 80% sensitivity and >70% accuracy in distinguishing gastric cancer from controls. Prospectively, both markers reached 87.5% sensitivity and 100% specificity, with high NPV and low negative likelihood ratio, supporting their potential for early screening. CFL1 showed higher disease specificity and pre-analytical stability than TAGLN2 across multiple non-gastric cancers and special-condition samples. **Conclusions**: A combined panel with routine markers improved calibration and decision-curve benefit, suggesting that multi-marker testing can enhance risk stratification and screening efficiency.

## Simple Summary

Gastric cancer is often difficult to detect at an early stage, when treatment is more likely to work. This study explores whether two proteins that can be measured in blood, Cofilin-1 and Transgelin-2, could help find gastric cancer in a less invasive way. The authors tested these proteins in samples from people with gastric cancer, healthy people, people with non-cancerous diseases or special clinical conditions, and people with other cancers. They also checked the results in both previously collected samples and newly collected samples. The findings suggest that these proteins may help separate gastric cancer from non-gastric conditions and could improve early detection. This work may give researchers practical blood-based markers to test in larger studies and support the development of easier tools for gastric cancer screening and diagnosis.

## 1. Introduction

Gastric cancer (GC) presently occupies the fifth position in global cancer incidence and the fourth in fatality rates. The incidence escalates with age, and there is a tendency for younger demographics to be impacted [1,2]. Gastric cancer has several histological forms, with adenocarcinoma as the predominant variant, often linked to *Helicobacter pylori* infection [3]. The pathophysiology of gastric cancer is intricate, with *Helicobacter pylori* infection—a Group 1 carcinogen—strongly linked to non-cardia gastric cancer. Various factors, such as high-salt diets, smoking, alcohol consumption, gastrointestinal dysbiosis, obesity, and genetic predisposition, together contribute to a multistage pathogenic process. This process entails the prolonged inflammation of normal gastric mucosa, leading to atrophy, intestinal metaplasia, and eventually malignant transformation [4]. The Epstein–Barr virus infection is a notable contributor to gastric cancer, responsible for roughly 10% of cases. It is more common in male patients and in the gastroesophageal junction subtype. EBV facilitates gastric carcinogenesis by enhancing hypermethylation of tumor suppressor genes, inciting gastric mucosal inflammation, and enabling immune evasion by the host [5]. Co-infection with *Helicobacter pylori* and Epstein–Barr virus may elevate the risk of gastric cancer formation and enhance its invasiveness due to the chronic inflammation induced by both pathogens in the gastric. Advancements in medical technology have led to a reduction in global incidence and fatality rates for gastric cancer. The worldwide burden of gastric cancer is anticipated to rise by 62% by 2040 [6,7].

Initial tumor biomarkers predominantly depended on protein markers found in bodily fluids or tissues, including hormones, enzymes, and antigens, with detection frequently utilizing immunological techniques such as radioimmunoassay. Advancements in detection technology, including mass spectrometry, gene chips, and high-throughput sequencing, now provide a thorough assessment of tumor features at several levels, encompassing DNA, RNA, proteins, and metabolites [8]. Immunohistochemistry and PCR, due to their operational simplicity and cost-effectiveness, continue to be prevalent techniques for protein and gene analysis in clinical settings. Conversely, next-generation sequencing facilitates extensive examination of various genomic alterations—including mutations, gene fusions, and copy-number variations—via massively parallel sequencing, and has proven to be particularly valuable in solid tumors such as non-small cell lung cancer, colorectal cancer, and melanoma [9]. Serum tumor markers are extensively utilized in cancer diagnosis and therapy, primarily for early screening, supplementary diagnosis, prognostic evaluation, evaluating treatment efficacy, and surveillance for cancer recurrence. In the realm of early cancer screening, liquid biopsy has garnered heightened interest as a non-invasive and economical testing method. Liquid biopsy presents novel opportunities for the early diagnosis, treatment monitoring [10], and prognostic evaluation of gastric cancer by identifying tumor biomarkers in blood, including circulating tumor cells [8], circulating tumor DNA, DNA methylation, and non-coding RNA [8,11,12,13]. Liquid biopsies have advantages over standard tissue biopsies, including minimally invasive techniques, cost-effectiveness, and time efficiency. They permit the collection of several samples at predetermined intervals, thereby allowing the observation of dynamic tumor alterations. About 40% of gastric tumors demonstrate amplification of receptor tyrosine kinase genes, including EGFR, ERBB2, ERBB3, FGFR2, JAK2, and MET, in conjunction with mutations in KRAS or NRAS, cell cycle regulators, and VEGFA [1,14]. These signaling molecules and pathways are crucial in the initiation, development, metastasis, and therapeutic response of gastric cancer, rendering them potential biomarkers and therapeutic targets for the disease. Gastric cancer consists of four molecular subtypes: EBV, MSI, chromosomal instability, and genomic stability. The molecular subtypes are significantly correlated with patient prognosis; specifically, individuals with MSI and EBV subtypes demonstrate more favorable results post-surgery, whereas those with the genomic stability subtype exhibit the least favorable prognosis [15]. In addition to biomarkers utilized for early screening and diagnosis, the advancement of prognostic biomarkers allows doctors to evaluate disease progression risk and therapeutic response with greater precision. In tumor immunotherapy, immune-related indicators such as tumor mutational burden, microsatellite instability, and tumor-infiltrating lymphocytes are essential for assessing patient responses to immune checkpoint inhibitors. Biomarker testing aids in the risk categorization of patients during tumor prognostic evaluation.

Nonetheless, the clinical utilization of biomarkers continues to encounter several obstacles. Conventional serum markers are challenging to utilize independently in tumor serum diagnosis due to limitations, including inadequate specificity and vulnerability to influence from non-tumor variables [16]. Many early cancer patients have false negative results in traditional tumor marker testing, which is not conducive to cancer screening and early intervention. When patients experience physical discomfort and are diagnosed with cancer through pathological biopsy, local lymph node or distant metastasis often occurs. For instance, alpha-fetoprotein (AFP), carcinoembryonic antigen (CEA), carbohydrate antigen CA199, and CA125 assays exhibit insufficient sensitivity in individuals with early-stage gastric cancer. Moreover, there are currently no particular serum tumor markers for gastric cancer, complicating early screening and detection. In recent years, artificial intelligence and multi-omics research have offered novel insights for tumor marker diagnoses [17,18,19,20,21]. This study expands on our previous multi-omics and machine learning research on early gastric cancer by assessing the diagnostic efficacy of novel gastric cancer tumor markers through both prospective and retrospective cohort studies [22], providing new insights for early screening and in vitro diagnosis of gastric cancer.

## 2. Materials and Methods

### 2.1. Screening Diagnostic Markers for Early Gastric Cancer Using Serum Proteomics

The non-metastatic gastric cancer patients (GCMN group) and healthy controls (HC group) were subdivided into three subgroups each (GCMN-1, GCMN-2, GCMN-3, and HC-1, HC-2, HC-3); each GCMN subgroup comprised 10 serum samples, whereas the HC subgroups contained 10, 10, and 9 serum samples, respectively. Samples from each subgroup were aggregated, digested, desalted, and fractionated. Proteins were digested utilizing a Nomimicro/general-purpose protein digestion kit (Naomi MagicOmics-MMB8X, Suzhou, China): 20 μL of protein solution was introduced to wells containing MMB beads and incubated at 37 °C for 30 min. Subsequently, 45 μL of binding buffer was incorporated, and the mixture was incubated at room temperature with gentle agitation for 15 min. The supernatant was thereafter discarded, and the MMB beads were rinsed three times with washing buffer. Subsequently, 20 μL of the digestion working solution was added to resuspend the beads, followed by incubation at 37 °C for a minimum of 4 h. The reaction was concluded by the addition of 5 μL of quenching buffer, followed by lyophilization of the material. Samples were labeled, dissolved in 100 μL of mobile phase A, centrifuged at 14,000× *g* for 20 min, and the supernatant was collected for high-performance liquid chromatography (HPLC) separation at a flow rate of 0.7 mL/min. A spectrum library was created for pooled samples from each subgroup in the GCMN and HC groups for serum proteome analysis utilizing data-dependent acquisition (DDA) mode. Mobile phases A (100% water, 0.1% formic acid) and B (80% acetonitrile, 0.1% formic acid) were formulated; lyophilized samples were reconstituted in 10 μL of mobile phase A, subjected to centrifugation at 14,000× *g* for 20 min at 4 °C, and 400 ng of the resulting supernatant was injected for analysis. A timsTOF_HT mass spectrometer with a CaptiveSpray ion source was utilized for DDA analysis, spanning a mass range of 300–1500 *m*/*z*. The first mass spectrometry resolution was established at 60,000 (1222 *m*/*z*) with an accumulation duration of 100 ms in the TIMS tunnel, a capillary voltage of 1.6 kV, and a mobility range of 0.6–1.6 cm^2^/(V·s). The whole cycle duration was 1.1 s, comprising 10 PASEF cycles, with a separation flow rate of 500 nl/min. Each sample was analyzed separately using data-independent acquisition (DIA), adhering to the same initial protocol as for data-dependent acquisition (DDA); in DIA mode, the accumulation time was 50 ms, the capillary voltage was 1.5 kV, the mobility range was 0.70–1.30 cm^2^/(V·s), the total cycle time was 1.23 s, and the flow rate was 300 nl/min. Data integration was ultimately executed utilizing analytical software to derive quantitative values for serum proteins.

The mass spectrometry proteomics data have been submitted to the ProteomeXchange Consortium (http://proteomecentral.proteomexchange.org (accessed on 31 May 2025)) through the iProX partner repository, with the dataset number PXD061032 [23,24].

### 2.2. Clinical Serum Sample Collection and Inclusion Criteria

This study categorizes GCMN (T1-4N0M0) as early-stage gastric cancer. For the retrospective study, we gathered serum samples from 176 patients with non-metastatic gastric cancer between April and September 2025, along with 88 healthy control serum samples, and serum samples from seven additional non-metastatic cancer types: 18 patients with colorectal cancer, 22 patients with liver cancer, 23 patients with lung cancer, 12 patients with prostate cancer, 5 patients with breast cancer, 5 patients with cervical cancer, and 9 patients with esophageal cancer. For the prospective study, we collected serum samples from 88 patients referred to the Gastroenterology Department, whose gastric cancer status had not yet been determined at the time of blood collection. Concurrently, serum samples were also collected from three patients with a confirmed diagnosis of early gastric cancer and three healthy controls for comparative analysis. Upon verifying the pathological diagnoses of the patients, we quantified the individuals diagnosed with gastric cancer and juxtaposed this data with the results of the ELISA test. All participants satisfied the subsequent criteria: Oncology patients: (1) Histopathological diagnosis; (2) Medical imaging diagnostic; (3) Age of 18 years or older; (4) Conducted serum tests for conventional tumor markers (CEA, AFP, CA199, CA125), full blood count, and pertinent pathological diagnosis. Patients with gastric cancer were endoscopically verified to be free of esophageal or duodenal ulcers and other abnormalities. Individuals without health conditions: (1) Absence of familial cancer history or prior diagnosis/treatment of gastric disease; (2) Absence of benign gastric ulcers, benign gastric neoplasms, gastric lymphoma, gastrointestinal stromal tumors, or gastric neuroendocrine tumors; (3) Normal complete blood count. All blood samples were procured in serum separation tubes with a clot activator to yield serum. Before sampling, all cancer patients had not had chemotherapy, radiation, or surgical intervention. Individuals with autoimmune disorders, infectious illnesses, or multiple malignancies were likewise eliminated. The staging of gastric cancer was categorized according to the 8th edition TNM classification system of the Union for International Cancer Control (UICC).

Conditions of serum samples may compromise test outcomes. To replicate authentic clinical circumstances, we gathered specialized serum specimens from non-cancer patients with various diseases and healthy persons as the experimental group, utilizing a healthy control group for biomarker performance validation. The sample comprised serum from 9 patients with infectious diseases, 8 patients with autoimmune diseases, 9 patients with gastric ulcers, 5 patients with hyperbilirubinemia, 5 patients with hyperlipidemia, and 4 patients with hemolysis. All procedures involving human subjects in this study received approval from the Research Ethics Committee of Lanzhou University Second Hospital (Approval No. 2023A-459).

### 2.3. Enzyme-Linked Immunosorbent Assay (ELISA)

Prior to the experiment, equilibrate the kit and serum samples at ambient temperature for 15 min. Thereafter, reconstitute the lyophilized standards using the universal diluent. Conduct consecutive twofold serial dilutions of TAGLN2 (0–50 ng/mL) and CFL1 (0–10 ng/mL) to establish standard wells for detection. Serum samples were diluted at specified ratios (TAGLN2 1:100, CFL1 1:5) to mitigate matrix effects. Introduce 100 µL of standards or diluted serum into pre-coated microplates and incubate at 37 °C for one hour. The CFL1 plate was thereafter washed three times with 300 µL of wash buffer; this procedure was excluded for the TAGLN2 plate. Subsequently, 100 µL of 1 × biotinylated antibody was added (37 °C, 1 h), followed by 100 µL of 1× enzyme conjugate (37 °C, 30 min). The TAGLN2 plate underwent three washes, while the CFL1 plate underwent four washes. During the color development phase, include 50 µL of substrates A and B into CFL1, and 90 µL of substrate into TAGLN2. Incubate at 37 °C in the absence of light for 15 min. Cease the reaction by adding 50 µL of stop solution and promptly measure the optical density (OD) at 450 nm. Construct a four-parameter logistic function standard curve utilizing the standard well concentrations and the recorded optical density values. Standard curves with coefficients of determination (R^2^) > 0.99 were considered acceptable. The four-parameter logistic curve function of the ELISA standard wells is shown in Equation (1):

(1) $$y\;=\;\frac{A\;-\;D}{[1\;+\;({\frac{x}{C})}^{B}]}\;+\;D$$

Among them, *x* is the concentration of the standard, *y* is the corresponding OD value, *A* and *D* are the upper and lower asymptotes of the curve, respectively, *C* is the concentration corresponding to the midpoint of the curve (EC50), and *B* is the slope of the curve (Hill coefficient).

The CFL1 ELISA kit (YJ060405/96T) was purchased from Shanghai Yuanju Biotechnology Center (Shanghai, China), and the TAGLN2 ELISA kit (JL47102/96T) was purchased from Shanghai Jianglai Biotechnology Co., Ltd. (Shanghai, China). The intra- and inter-assay coefficients of variation (CVs) for the ELISA were kept below 10% and 10%, respectively. Patient data for conventional tumor markers AFP, CEA, CA199 and CA125 were obtained from serum sample analyses conducted with the Roche Cobas p501 chemiluminescence immunoassay analyzer in our laboratory. The maximum threshold of our laboratory’s reference range for each traditional tumor marker served as the cutoff value. When the concentration of the biomarker reaches or exceeds the cutoff, the test result is defined as positive; When it is below the cutoff, the test result is defined as negative. Subsequently, match the ELISA detection results with the pathological examination results to assess the diagnostic performance of the biomarkers.

### 2.4. Construction of Nomogram for Gastric Cancer Diagnosis Model

Create a numerical matrix with the blood biomarker protein concentration values for each patient with gastric cancer and healthy controls. If the average concentration of each biomarker in all samples is greater than 1 ng/mL, then exclude samples with low biomarker expression. To identify grouping, read the lists of gastric cancer and healthy control samples using R 4.5.0. Before merging the data for modeling, encode the outcome variable Type as either non-gastric cancer (HC = 0) or gastric cancer (GC = 1). Fit a binary logistic regression model after choosing biomarkers for analysis, then take the model summary and extract the regression coefficients and their statistical significance. The logistic regression model’s projected likelihood of “developing gastric cancer (Type = 1)” is the individual risk score. The linear predictor (*LP*) was first determined, as shown in Equation (2):

(2) $$\mathrm{LP}\;=\;\beta_{0}\;+\;\sum_{i=1}^{k}\beta_{i}X_{i}$$

where *X_i_* represents the concentration of each serum biomarker, and *β_i_* denotes the corresponding regression coefficient. Subsequently, the Logistic function is applied to convert these values into probability *P*. Based on this, the linear predicted values are mapped to cancer risk probabilities and plotted as a histogram. The risk score was calculated as shown in Equations (3) and (4):

(3) $$\mathrm{Riskcore}\;=\;P\;(\mathrm{Type}\;=\;1)\;=\;\frac{1}{(1\;+\;\exp\left(-\mathrm{LP}\right))}$$

(4) $$\exp(-\mathrm{LP})=e^{-\mathrm{LP}}$$

where the natural exponential function is shown by exp. *P* gets closer to 1 as the *LP* value rises. The accuracy of the probability scale is assessed by plotting the “predicted probability versus actual probability” calibration curve using the bootstrap technique calibrate (method = “boot”, B = 1000). The decision_curve and plot_decision_curve functions are used to perform decision curve analysis at thresholds between 0 and 1.

### 2.5. Comparison of Diagnostic Performance of Biomarkers

Based on the results of the novel marker ELISA experimental detection and the previous clinical detection of traditional tumor biomarkers, Graphpad Prism 8.0.1 software was used to plot and calculate the independent ROC curves and AUC values for six tumor markers. The combined marker diagnostic score for each subject was calculated according to the linear prediction expression of the logistic regression model. The calculation formula is: Combined diagnostic score = Intercept + *β*_1_ × biomarker 1 concentration + *β*_2_ × biomarker 2 concentration + … + *β*_6_ × biomarker 6 concentration. Here, Intercept is the intercept term, and *β*_1_–*β*_6_ are the regression coefficients corresponding to each biomarker in the diagnostic model. After converting the serum concentrations of each marker into diagnostic scores, the ROC curves and AUC values for the combination of multiple tumor markers were plotted and calculated according to different groupings.

### 2.6. Stratified Expression and T-Category Association Analysis of Novel Biomarkers

The TCGA-STAD cohort (https://portal.gdc.cancer.gov/) (accessed on 9 May 2026) was used to analyze the expression patterns of CFL1 and TAGLN2 across clinicopathological and molecular subgroups of gastric cancer. RNA-seq TPM expression data were used to extract the expression values of CFL1 and TAGLN2. The first 12 characters of each TCGA sample barcode were used as the patient identifier and matched with the clinical annotation file. Gene expression values were transformed as log_2_(TPM + 1) for subsequent analyses. For patients with multiple primary tumor samples, the average expression value was used as the patient-level expression level.

Lauren histological classification was derived from the histological type annotation in the clinical file. Cases annotated as intestinal adenocarcinoma were classified as intestinal type, whereas those annotated as diffuse type were classified as diffuse type. Adenocarcinoma NOS, other histological types, and cases with missing histological annotation were excluded from the main intestinal-versus-diffuse comparison. Differences in CFL1 and TAGLN2 expression between intestinal-type and diffuse-type tumors were assessed using the Wilcoxon rank-sum test. TCGA molecular subtype annotation was obtained from the TCGA gastric cancer molecular subtype dataset, including EBV-positive, microsatellite instability, genomically stable, and chromosomal instability subtypes, corresponding to Epstein–Barr virus (EBV), Microsatellite instability (MSI), Genomically stable (GS), and Chromosomal instability (CIN), respectively [25]. This analysis included only samples with both RNA-seq expression data and available molecular subtype annotation. Overall differences in CFL1 and TAGLN2 expression among the four molecular subtypes were assessed using the Kruskal–Wallis test. When a significant overall difference was observed, pairwise Wilcoxon rank-sum tests were performed with Benjamini–Hochberg correction for multiple comparisons. Pathological T categories were extracted from the clinical staging annotation, with a focus on T1a, T1b, and T2; T2 included T2, T2a, and T2b. Differences among these three groups were assessed using the Kruskal–Wallis test, and Spearman correlation analysis was used to evaluate the trend between gene expression and increasing early T category. In addition, T1, T1a, and T1b were combined as T1, while T2, T2a, and T2b were combined as T2 for a T1-versus-T2 sensitivity analysis. Meanwhile, one-way analysis of variance (ANOVA) was performed to stratify the clinical gastric cancer patients included in the retrospective study according to tumor TNM stage. After matching these groups with the biomarker ELISA results, intergroup differences were analyzed. The data analysis in this section is conducted using R 4.5.0 software.

### 2.7. Data Processing and Statistical Analysis

Volcano plot and heatmap were plotted by https://www.bioinformatics.com.cn (last accessed on 1 April 2025), an online platform for data analysis and visualization [26]. Proteins with a fold change (FC) absolute value |FC| ≥ 1.5 and *p* < 0.05 were deemed to be significantly differentially expressed between the GCMN and HC groups in serum proteomics analysis. Based on the absorbance values and matching concentrations of the standard samples, a standard curve was created for the ELISA experiment using the four-parameter logistic function in the ELISA Calc version 0.1 software. The relevant serum protein concentrations are calculated by entering the OD values from the sample wells into the curve equation. Mean ± standard deviation (SD) is used to express the results. Differential analysis, box plots, and ROC curves were produced using GraphPad Prism 8.0.1 software. The Kruskal–Wallis test was used for comparisons between many groups, while the Mann–Whitney U test was employed for comparisons between two groups because of the non-normal distribution and unequal variance. The threshold for statistical significance was set at *p* < 0.05. Clinical sensitivity (CSE), clinical specificity (CSP), positive likelihood ratio (+LR), negative likelihood ratio (−LR), positive predictive value (PPV), negative predictive value (NPV), diagnostic accuracy, and false negative rate were among the biomarker’s performance evaluation metrics. The following calculation techniques are described in detail in Table 1:

CSE = a/(a + c) × 100%CSP = d/(b + d) × 100%+LR = CSE/(1 − CSP)−LR = (1 − CSE)/CSPPPV = a/(a + b) × 100%NPV = d/(c + d) × 100%Diagnostic Accuracy = (a + d)/(a + b + c + d) × 100%False Negative Rate = c/(a + c) × 100%

**Table 1:** Receiver operating characteristic curve for evaluating diagnostic efficiency of biomarker concentrations in gastric cancer.

Biomarker Concentration (ng/mL)	Clinically Confirmed Cases		
	Gastric Cancer	Non-Gastric Cancer	Total
Positive (≥cut off)	a	b	a + b
Negative (<cut off)	c	d	c + d
Total	a + c	b + d	a + b + c + d

Whereas NPV shows the likelihood that a negative test result actually rules out gastric cancer, PPV shows the likelihood that a positive test result actually reveals gastric cancer. They vary according to the occurrence of gastric cancer and collectively establish the dependability of a single test result. The positive likelihood ratio (+LR) was calculated as CSE divided by the false-positive rate, namely CSE/(1 − CSP), and reflects how much a positive biomarker result increases the probability of gastric cancer. The negative likelihood ratio (−LR) was calculated as the false-negative rate divided by CSP, namely (1 − CSE)/CSP, and reflects how much a negative biomarker result decreases the probability of gastric cancer. Unlike PPV and NPV, likelihood ratios are less affected by disease prevalence and can be used to estimate post-test probability from pre-test probability. Both depend only on the intrinsic accuracy of the test and are not influenced by population prevalence. To help with the diagnosis of gastric cancer, they can be used to compute posterior probabilities under various prior probabilities. Consequently, a thorough evaluation of a biomarker’s diagnostic efficacy is made possible by integrating these evaluation factors in clinical practice, which also maximizes individualized gastric cancer diagnosis and treatment plans.

The required sample size for the case-control study comparing gastric cancer patients with healthy controls using ELISA testing was calculated as shown in Equation (5):

(5) $$n\;=\;\frac{(1\;+\;\frac{1}{k})\cdot (Z_{\alpha /2}\;+\;Z_{\beta }{)}^{2}\cdot \sigma^{2}}{\Delta^{2}}$$

where *k* is the ratio of sample sizes between the case group and the control group, and n is the total sample size needed. Z_β_ is the critical number for statistical power (1 − β), while Z_α/2_ is the crucial value for the two-tailed significance level α. Z_α/2_ = 1.96 and Z_β_ = 1.28 when α = 0.05 and β = 0.1. *σ* is the pooled SD of serum protein concentrations (ng/mL) between the control and case groups for the biomarker under study, and Δ is the mean difference in serum protein concentrations between these groups. The pooled standard deviation (SD) was calculated as shown in Equation (6):

(6) $$\sigma \;=\;\sqrt{\frac{\left(n_{1\;}-\;1\right)s_{1}^{2}\;+\;(n_{2}\;-\;1)s_{2}^{2}}{n_{1}\;+\;n_{2\;}-\;2}}$$

where *s*_1_ and *s*_2_ stand for the case group and control group SD, respectively, and *n*_1_ and *n*_2_ represent the sample sizes of the case group and control group, respectively. The proportion of each group’s actual sample size to the overall sample size is multiplied by the total sample size to determine the theoretical required sample size. The sample size needed to get the appropriate statistical power for the assay can be ascertained by statistically analyzing the protein concentration results for each sample after ELISA testing.

## 3. Results

### 3.1. Potential Biomarkers CFL1 and TAGLN2 Were Identified Through Serum Proteomics and Machine Learning Analysis

**Figure 1:**
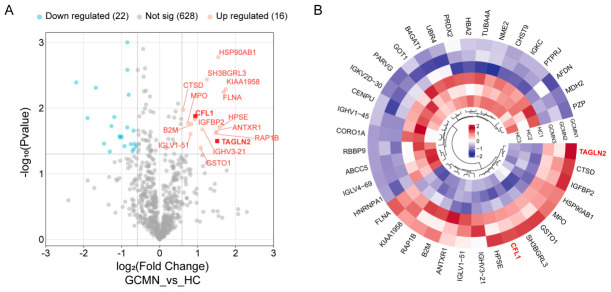
Serum proteomics identification of differential proteins between non-metastatic gastric cancer patients and healthy controls. (**A**) A total of 666 serum proteins were identified between the two groups, with 16 proteins showing upregulation and 22 downregulation in the gastric cancer group. (**B**) Heatmap of differentially expressed proteins between the two groups. Based on prior research, CFL1 and TAGLN2 were ultimately selected as potential diagnostic biomarkers for gastric cancer, and their clinical performance was validated.

Our preliminary investigation revealed that serum proteomics identified 666 proteins common to both the GCMN group and the HC group (Figure 1A). In comparison to the HC group, the GCMN group exhibited 16 upregulated proteins and 22 downregulated proteins. A heatmap illustrates the gene names associated with each differentially expressed protein (Figure 1B). According to machine learning and qRT-PCR findings from previous studies [22], CFL1 and TAGLN2 exhibited elevated expression levels in the blood and cancerous tissues of gastric cancer patients. This study identified CFL1 and TAGLN2 as prospective diagnostic biomarkers for gastric cancer to assess their clinical efficacy and develop a diagnostic model.

### 3.2. Retrospective Analysis Indicates That the Novel Biomarkers CFL1 and TAGLN2 Demonstrate Good Diagnostic Sensitivity and Disease Specificity for Gastric Cancer

**Figure 2:**
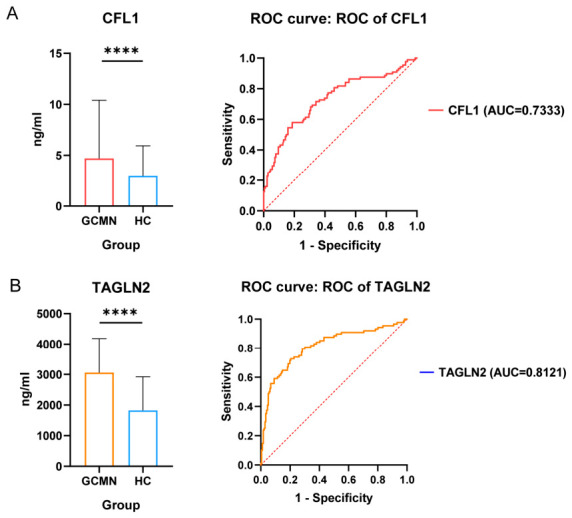
Retrospective analysis of serum CFL1 and TAGLN2 concentration differences between gastric cancer patients and healthy controls with ROC curve. (**A**) Analysis of serum concentration differences and ROC curve for CFL1. (**B**) Analysis of serum concentration differences and ROC curve for TAGLN2. (**** *p* < 0.0001).

The requisite sample size for the calculation was determined by substituting the CFL1 and TAGLN2 concentration values from each sample acquired through ELISA testing. The findings demonstrate that with α = 0.05 and β = 0.1, the sample size is sufficient and satisfies the statistical power criteria for a case-control study (Table 2). Consequently, the sample size utilized in this investigation offers adequate support for the research conclusions. Figure S1 illustrates the standard curve for the ELISA assay in the retrospective investigation. Differential analysis indicated that serum levels of CFL1 and TAGLN2 were markedly elevated in gastric cancer patients relative to healthy controls (*p* < 0.05). Figure 2 presents the findings of the differential analysis between the gastric cancer group and healthy controls, as well as the ROC curves for CFL1 and TAGLN2. The concentration associated with the peak Yodan index (Yodan index = CSE + CSP − 1) was utilized as the biomarker cutoff value for the retrospective analysis. The retrospective evaluation parameters for the biomarkers CFL1 and TAGLN2 were computed, with findings displayed in Table 3. CFL1 and TAGLN2 demonstrated CSE values approximately 80%, diagnostic accuracy beyond 70%, and minimal −LR. This indicates that CFL1 and TAGLN2 demonstrate strong diagnostic efficacy in differentiating early gastric cancer patients from healthy persons. The ROC curve performance parameters of biomarkers in retrospective analysis are shown in Table S1.

**Figure 3:**
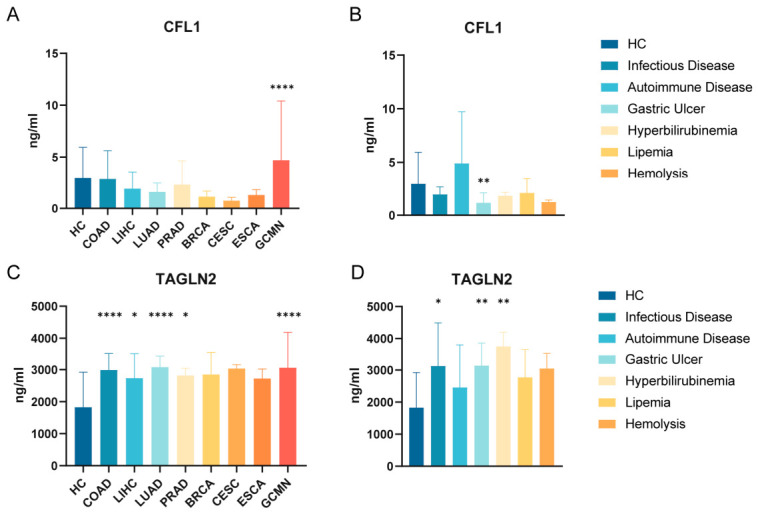
Specificity analysis of CFL1 and TAGLN2 in eight cancer and non-cancer diseases. (**A**) Analysis of CFL1 concentration differences between 8 cancer types and healthy controls. (**B**) Analysis of CFL1 concentration differences between infectious disease, autoimmune disease, and gastric ulcer patients, as well as 3 special condition samples and healthy controls. (**C**) Analysis of TAGLN2 concentration differences between 8 types of cancer and healthy controls. (**D**) Analysis of TAGLN2 concentration differences between infectious diseases, autoimmune diseases, gastric ulcer patients, and 3 special condition samples versus healthy controls. (* *p* < 0.05, ** *p* < 0.01, **** *p* < 0.0001) COAD, Colon adenocarcinoma; LIHC, Liver hepatocellular carcinoma; LUAD, Lung adenocarcinoma; PRAD, Prostate adenocarcinoma; BRCA, Breast invasive carcinoma; CESC, Cervical squamous cell carcinoma and endocervical adenocarcinoma; ESCA, Esophageal carcinoma.

Disease-specific analysis indicated no substantial disparity in serum CFL1 levels between patients with seven non-gastric malignancies (excluding gastric cancer) and healthy persons (Figure 3A), hence exhibiting commendable disease specificity. Serum CFL1 levels exhibited no significant difference between special-state samples from healthy individuals and healthy controls, but were decreased in patients with gastric ulcers (Figure 3B). Serum concentrations of TAGLN2 were markedly elevated in patients with colorectal, liver, lung, and prostate cancers compared to healthy individuals (Figure 3C). TAGLN2 levels were dramatically increased in hyperbilirubinemic blood and much higher in patients with infectious diseases and gastric ulcers compared to healthy individuals (Figure 3D). The data demonstrate that CFL1 shows significant specificity for gastric cancer compared to other malignancies, with detection being consistent regardless of sample status, leading to enhanced diagnostic stability and reliability.

### 3.3. Prospective Analysis Indicates That CFL1 and TAGLN2 Demonstrate Promising Performance in Early Gastric Cancer Screening

Early gastric cancer presents with insidious symptoms, and by the time patients experience discomfort, the malignancy has frequently penetrated the serosal layer or metastasised to adjacent lymph nodes. Consequently, efficient screening for gastric cancer tumor markers can swiftly identify cancer risk and enhance patient prognosis. We performed ELISA assays on serum samples from 88 individuals in the gastrointestinal outpatient clinic to quantify serum concentrations of CFL1 and TAGLN2. All patients lacked a confirmed diagnosis before the trial commenced. According to the ELISA findings from this prospective study, biomarker cutoff values were determined to differentiate gastric cancer from healthy subjects. Serum concentrations over the threshold amount were deemed indicative of gastric cancer. The standard curve from the prospective ELISA analysis is illustrated in Figure S2.

**Figure 4:**
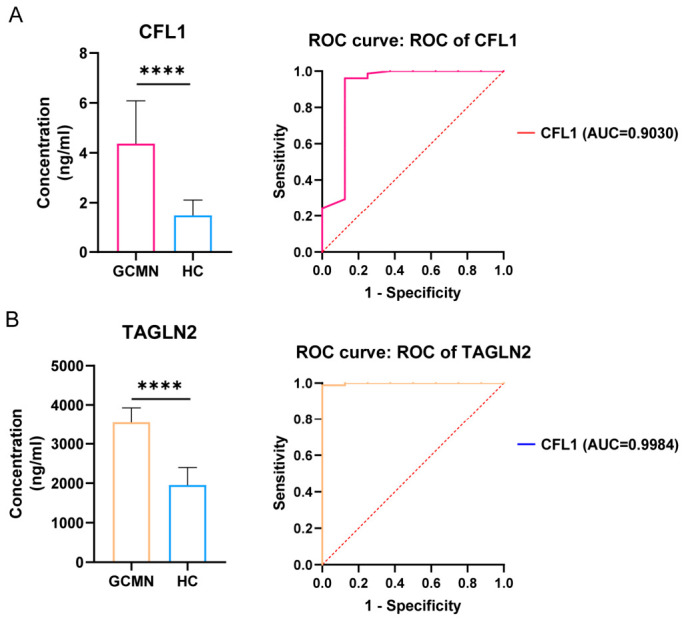
Prospective analysis of serum CFL1 and TAGLN2 concentration differences between gastric cancer patients and healthy controls with ROC curve. (**A**) Analysis of serum concentration differences and ROC curve for CFL1. (**B**) Analysis of serum concentration differences and ROC curve for TAGLN2. (**** *p* < 0.0001).

The subsequent pathology diagnostic verified gastric cancer in 5 out of 88 patients. According to the cutoff value, both CFL1 and TAGLN2 identified four positive cases in the prospective ELISA analysis, with the sole undiscovered case being the same individual. None of the remaining eight non-gastric cancer patients had a positive test result for CFL1. Neither marker yielded false positives. Serum test results for three confirmed gastric cancer patients and healthy individuals all surpassed the cutoff value, with serum diagnostic findings aligning with the real pathology diagnosis. The anticipated biomarker assessment criteria for CFL1 and TAGLN2 are detailed in Table 4, whilst the prospective differential analysis and ROC curves are illustrated in Figure 4. The +LR was incalculable due to a 100.00% CSP. The results indicated that CFL1 and TAGLN2 attained 87.5% CSE and 100.00% CSP in prospective analysis, showcasing elevated NPV, diagnostic accuracy, and low −LR. This suggests that CFL1 and TAGLN2 can facilitate early detection of gastric cancer in patients. The prospective and retrospective evaluations of biomarker performance for CFL1 and TAGLN2 indicate that these biomarkers exhibit dependable diagnostic efficacy in gastric cancer and facilitate early screening concurrently. Significantly, in contrast to TAGLN2, CFL1 demonstrates greater specificity for gastric cancer and is less influenced by the condition of serum samples. The ROC curve performance parameters of the novel biomarkers in the prospective analysis are shown in Table S2.

### 3.4. Development and Comparison of Gastric Cancer Diagnostic Models Using Traditional and Novel Tumor Biomarkers

To enhance the evaluation of the diagnostic efficacy of novel biomarkers and their superiority over conventional markers in serum-based gastric cancer detection, we developed a gastric cancer diagnostic model utilizing logistic regression. This model included serum concentrations of CFL1 and TAGLN2 from gastric cancer patients and healthy controls in a retrospective investigation, together with conventional markers AFP, CEA, CA199, and CA125. We assessed whether the model’s performance enhanced when utilizing novel biomarkers independently or in conjunction with traditional markers. Evaluation criteria were determined according to the threshold values for conventional tumor markers defined in our laboratory, with findings displayed in Table 5. The CSE of gastric cancer screening for both AFP and CEA was below 10%, with both markers demonstrating a miss rate for gastric cancer above 90%. With the exception of CEA, the diagnosis accuracy of the other three conventional tumor markers was below 60%. The −LR for all four conventional tumor markers in gastric cancer diagnosis exceeded those for CFL1 and TAGLN2. This suggests that negative outcomes for conventional tumor markers do not signify a reduced individual risk for gastric cancer and are ineffective in screening for early-stage gastric cancer patients.

**Figure 5:**
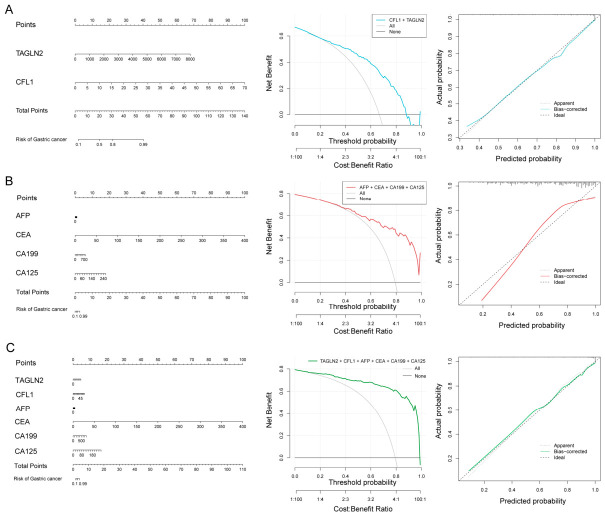
Diagnostic models for gastric cancer using novel tumor markers and traditional tumor markers. (**A**) Receiver operating characteristic curve, decision curve, and calibration curve for the gastric cancer diagnostic model using the novel biomarker combination. (**B**) Receiver operating characteristic curve, decision curve, and calibration curve for the gastric cancer diagnostic model using the traditional biomarker combination. (**C**) Receiver operating characteristic curve, decision curve, and calibration curve for the gastric cancer diagnostic model using both the novel and traditional biomarker combinations.

The diagnostic model’s nomogram, calibration curve, and decision curve for gastric cancer are shown in Figure 5. Increased levels of TAGLN2 and CFL1 indicators substantially influence gastric cancer outcomes (Figure 5A), while AFP and CA199 in the conventional marker combination exhibit a diminished impact on gastric cancer outcomes (Figure 5B). The calibrated curve of the conventional biomarker combination demonstrated a higher discrepancy between anticipated and actual values than the novel biomarker combination, signifying a diminished diagnostic efficacy for early gastric cancer (Figure 5B). The area beneath the decision curve for the combined biomarker panel surpassed that of the new biomarker panel or the traditional biomarker panel individually, demonstrating reduced discrepancies between projected and actual values on the calibration curve (Figure 5C). The novel biomarker panel exhibited enhanced predictive efficacy for early gastric cancer relative to the conventional biomarker panel.

### 3.5. Combined Detection of New and Traditional Tumor Markers Can Enhance the Diagnostic Efficacy of Early Gastric Cancer

**Figure 6:**
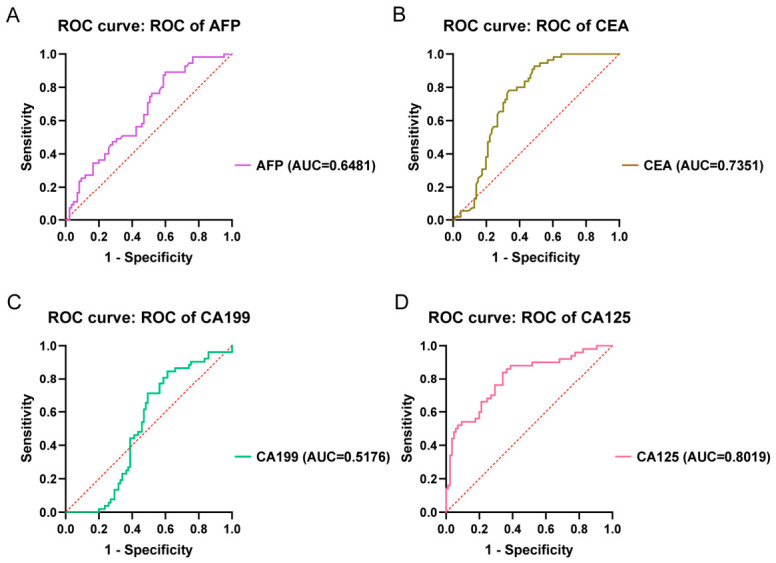
Retrospective analysis of ROC curves for traditional tumor markers in gastric cancer and healthy control groups. (**A**). Analysis of serum concentration differences and ROC curve for AFP. (**B**). Analysis of serum concentration differences and ROC curve for CEA. (**C**). Analysis of serum concentration differences and ROC curve for CA199. (**D**). Analysis of serum concentration differences and ROC curve for CA125.

The diagnostic ROC curves of traditional markers in the gastric cancer group are shown in Figure 6. The results indicate that the AUCs of AFP and CEA in early gastric cancer are both below 0.7 (Figure 6A,B), suggesting their overall discriminatory ability is limited. Although the AUCs of CEA and CA125 exceed 0.7 (Figure 6C,D), indicating a certain discriminatory ability, the CSE based on the current clinical cutoff values remains low, indicating that the thresholds of the existing clinical tumor markers CEA and CA125 are difficult to effectively distinguish between early gastric cancer patients and healthy individuals. Traditional tumor markers are difficult to achieve accurate screening for early gastric cancer while maintaining a low false positive rate.

**Figure 7:**
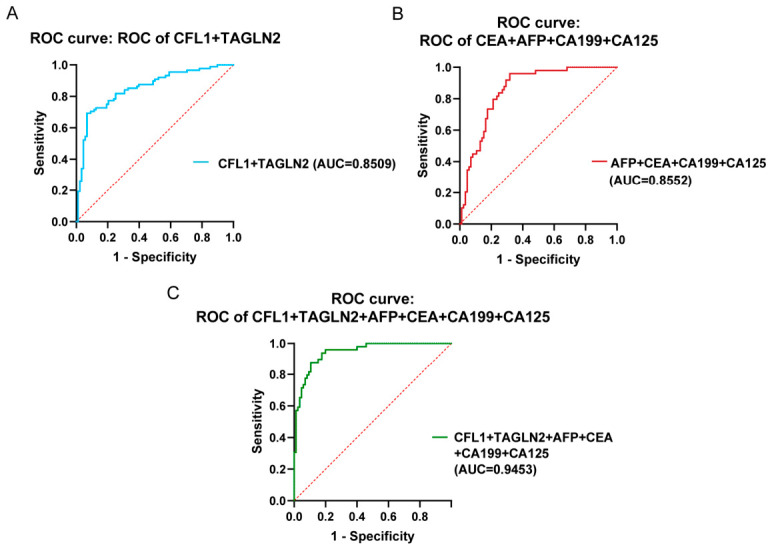
Retrospective analysis of ROC curves for traditional tumor biomarkers in gastric cancer and healthy control groups. (**A**). ROC curve of novel gastric cancer biomarkers CFL1 and TAGLN2 based on diagnostic model. (**B**). ROC curve of traditional tumor biomarkers CEA, AFP, CA199, and CA125 based on diagnostic model. (**C**). ROC curve of novel and traditional tumor biomarkers based on diagnostic model.

Based on the combined diagnostic scores of markers in three logistic regression models for gastric cancer, the ROC curves of combined tumor markers in the gastric cancer diagnosis model were constructed between the gastric cancer group and the healthy control group (Figure 7). The results showed that both the combination of CFL1 + TAGLN2 and the combination of the four traditional tumor markers exhibited AUC values close to 0.85 (Figure 7A,B), whereas the combination of the six new and old tumor markers showed a significantly improved AUC of 0.9453 (Figure 7C). Combining the diagnostic model with the ROC curve, it indicates that the predictive performance of the novel marker combination for gastric cancer is superior to that of the traditional marker combination, and the combined detection of novel and traditional biomarkers can significantly improve the model performance for early gastric cancer diagnosis. The ROC curve performance parameters of biomarker combinations in retrospective analysis are shown in Table S3.

### 3.6. Differential Expression of CFL1 and TAGLN2 in Different Molecular Subtypes and T Staging of Gastric Cancer

**Figure 8:**
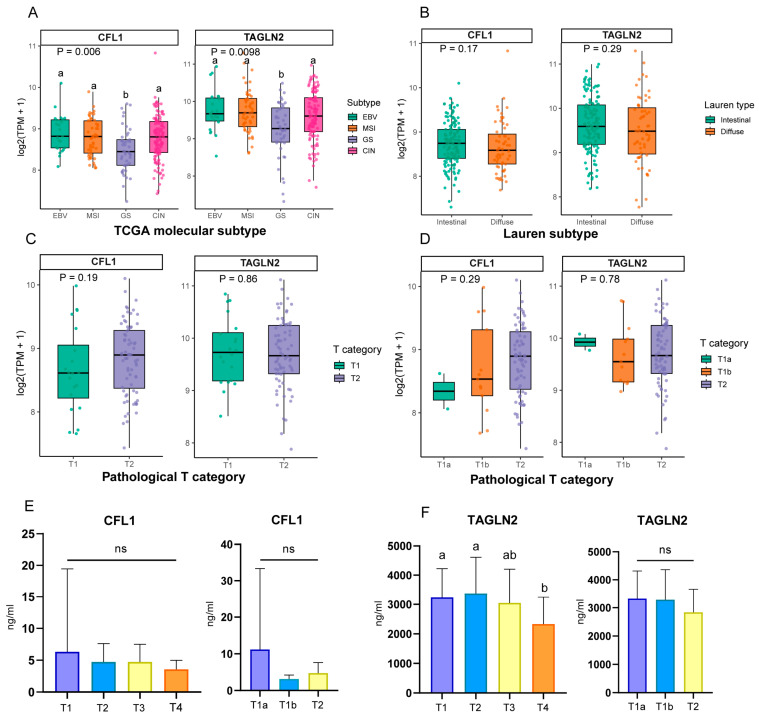
Differential expression analysis of CFL1 and TAGLN2 across histological subtypes, molecular subtypes, and early T categories. (**A**) Differential expression analysis of CFL1 and TAGLN2 among the four molecular subtypes, including EBV, MSI, GS, and CIN. (**B**) Differential expression analysis of CFL1 and TAGLN2 between intestinal-type and diffuse-type gastric cancer. (**C**) Differential expression analysis of CFL1 and TAGLN2 between T1 and T2 categories. (**D**) Differential expression analysis of CFL1 and TAGLN2 among T1a, T1b, and T2 categories. (**E**) Retrospective analysis of differences in serum CFL1 concentrations across T categories. (**F**) Retrospective analysis of differences in serum TAGLN2 concentrations across T categories. EBV, Epstein–Barr virus-positive; MSI, Microsatellite instability; GS, Genomically stable; CIN, Chromosomal instability. Different lowercase letters indicate significant differences between groups (*p* < 0.05). Groups sharing the same letter are not significantly different. The label “ab” indicates that the group is not significantly different from groups labeled “a” or “b”. “ns” indicates no significant difference.

In the TCGA-STAD cohort, the expression patterns of CFL1 and TAGLN2 were first evaluated across the four TCGA molecular subtypes. A total of 228 cases with available EBV, MSI, GS, or CIN molecular subtype annotation and matched RNA-seq expression data were included, consisting of 23 EBV, 49 MSI, 45 GS, and 111 CIN cases. Kruskal–Wallis analysis revealed significant differences in both CFL1 and TAGLN2 expression among the four molecular subtypes. Specifically, CFL1 and TAGLN2 expression levels were significantly higher in the EBV, MSI, and CIN subtypes than in the GS subtype, whereas no significant differences were observed among the EBV, MSI, and CIN subtypes (Figure 8A). The differential expression results of CFL1 and TAGLN2 across the four molecular subtypes are summarized in Table 6. CFL1 and TAGLN2 expression was further compared between intestinal-type and diffuse-type gastric cancers. This analysis included 143 intestinal-type and 59 diffuse-type cases, and no significant differences in CFL1 or TAGLN2 expression were observed between these two histological subtypes (Figure 8B).

The association between CFL1 and TAGLN2 expression and early pathological T categories was then assessed. The T1a, T1b, and T2 groups included 2, 13, and 72 cases, respectively. Neither CFL1 nor TAGLN2 showed a significant expression trend with increasing T category. Consistently, the combined T1-versus-T2 analysis also showed no significant association between CFL1 or TAGLN2 expression and early pathological T category (Figure 8C,D). Taken together, these results indicate that, in the TCGA-STAD cohort, CFL1 and TAGLN2 expression levels are mainly associated with the four TCGA molecular subtypes, but not with histological subtype or early pathological T category.

Based on the previously obtained ELISA results for the two candidate biomarkers and the corresponding clinical information, the serum concentrations of CFL1 and TAGLN2 in gastric cancer patients were further analyzed across different T categories. No significant differences in serum CFL1 concentration were observed among T1–T4 categories or among T1a, T1b, and T2 categories (Figure 8E). In contrast, serum TAGLN2 concentration differed significantly between T1 and T4, as well as between T2 and T4 gastric cancer cases (*p* < 0.05), whereas no significant difference was observed among T1a, T1b, and T2 categories (Figure 8F).

## 4. Discussion

Determining biomarkers and clarifying the molecular mechanisms underlying the complicated biological process of gastric carcinogenesis are essential for the early detection and management of gastric cancer. Currently utilized traditional serum tumor indicators exhibit inadequate performance for the early identification of gastric cancer, highlighting the necessity to identify biomarkers with enhanced sensitivity and specificity. In our initial study, we evaluated 6 prospective biomarkers—B2M, TAGLN2, CTSD, HSP90AB1, SH3BGRL3, and CFL1—and confirmed their gene expression levels in gastric cancer and surrounding non-tumor tissues using qRT-PCR. Four biomarkers (TAGLN2, HSP90AB1, SH3BGRL3, and CFL1) exhibited elevated expression levels in gastric cancer and were significantly correlated with immune cell infiltration in gastric tumors. This study identified CFL1 and TAGLN2 as promising biomarkers, assessing their diagnostic efficacy by ELISA-based serum measures and comparing it to a panel of four traditional tumor markers. The findings indicated that, in contrast to the four traditional tumor markers, CFL1 and TAGLN2 exhibited superior biomarker performance metrics in gastric cancer, facilitating the screening and identification of patients with early-stage gastric cancer.

Prior research has shown that TAGLN2 and CFL1 facilitate the onset and advancement of gastric cancer. TAGLN2 expression is exclusive to tumor cells, and the resultant Transgelin-2 modulates the myosin cytoskeleton, facilitates cell-cell adhesion, and augments cell motility [27,28]. It stimulates epithelial-mesenchymal transition (EMT) and facilitates gastric cancer metastasis through the PI3K/AKT or MAPK/ERK signalling pathways by promoting angiogenesis via the NRP1/VEGFR2-MAPK pathway [28,29]. As therapeutic targets, they can augment the lethal effects of CD8+ T lymphocytes by facilitating the development of immunological synapses between T lymphocytes and tumor cells [30,31]. CFL1 and TAGLN2 facilitate epithelial–mesenchymal transition (EMT) by reorganizing the actin cytoskeleton and modulating intercellular adhesion, hence imparting treatment resistance to gastric cancer cells. The resistance can be conveyed among gastric cancer cells through the PI3K-AKT-CFL1 signalling pathway or extracellular vesicles. In hepatocellular cancer, hypoxia-inducible factor-1α initiates CFL1 transcription by binding to hypoxia response sites inside its promoter. CFL1 inhibits ubiquitination to safeguard phospholipase D1 from degradation, thereby promoting epithelial–mesenchymal transition in hepatocytes.

This study found that the serum levels of TAGLN2 and CFL1 differed between gastric cancer patients and healthy persons, aligning with prior qRT-PCR findings. TAGLN2 demonstrated elevated serum levels in healthy subjects relative to CFL1. Nevertheless, the kit’s detection limit necessitating higher dilution factors may render TAGLN2 less feasible than CFL1 in clinical applications. Therefore, in the future, breakthroughs are needed in the development of reagent kits to reduce matrix effects caused by different dilution ratios of different detection indicators. TAGLN2 demonstrated increased expression in four non-gastric malignancies, with reduced disease specificity compared to CFL1. A thorough biomarker assessment demonstrated the enhanced clinical efficacy of CFL1 compared to TAGLN2. Figure 5 illustrates that the calibration curve of the CFL1-TAGLN2 combination diagnostic model closely corresponds with the ideal curve, while the calibration curves of the conventional four-tumor-marker models diverge from the ideal line. The calibration curve of the diagnostic model closely matched the ideal line when CFL1 and TAGLN2 were integrated with conventional markers. The decision curves for all three models indicated that the CFL1-TAGLN2 diagnostic model possesses greater practical clinical utility than conventional tumor markers. A thorough retrospective and prospective clinical validation of model predictions verifies that CFL1 and TAGLN2 are pivotal in identifying gastric cancer status inside the logistic regression model. Despite the testing of the two novel biomarkers in this study on various cancer samples, non-cancer samples, and specialized sample types, their analytical performance requires comprehensive validation for clinical chemiluminescence detection applications. Their specificity must guarantee the absence of cross-interference from other compounds in intricate clinical sample matrices, and their sensitivity must satisfy the threshold criteria for early illness diagnosis or trace biomarker identification. Simultaneously, standardized testing techniques and quality control protocols are vital, including uniform sample collecting and storage methods, standardized operational procedures, and the implementation of inter-batch and internal quality control measures. A thorough assessment of the test’s cost-effectiveness and accessibility is necessary to facilitate the translation of clinical use.

In the TCGA-STAD cohort, CFL1 and TAGLN2 expression differed significantly among the EBV, MSI, GS, and CIN molecular subtypes, with higher expression levels in the EBV, MSI, and CIN subtypes than in the GS subtype. In contrast, no significant association was observed with Lauren histological subtype or early pathological T category. These findings suggest that CFL1 and TAGLN2 may primarily reflect molecular heterogeneity rather than histological morphology or early local invasion depth in gastric cancer. Recent studies have also emphasized that TCGA molecular classification provides additional biological and prognostic information beyond Lauren classification, particularly for GS and CIN subgroups with distinct genomic features and clinical behavior [32]. The relatively lower expression of CFL1 and TAGLN2 in the GS subtype may therefore be related to biological processes enriched in non-GS subtypes, such as cytoskeletal remodeling, immune microenvironmental changes, or chromosomal instability. Recent multiomics and serum proteomic studies have also identified CFL1 and TAGLN2 as potential diagnostic biomarkers for gastric cancer and linked them to immune infiltration and early gastric cancer diagnostic models [22]. These observations support the subgroup-specific expression patterns of CFL1 and TAGLN2 identified in the present TCGA-STAD analysis.

In clinical serum samples, CFL1 concentrations did not differ significantly across T1–T4 categories or among T1a, T1b, and T2 groups. Serum TAGLN2 levels differed significantly between T1 and T4 as well as between T2 and T4, but not among T1a, T1b, and T2 categories. These findings suggest that serum TAGLN2 may be associated with more advanced local invasion or increased tumor burden, whereas its ability to distinguish early T subcategories remains limited. It should be noted that serum protein levels can be influenced by tumor release, host response, inflammatory status, sample handling, and assay platform, and therefore may not fully correspond to tumor tissue RNA expression. Recent reviews of gastric cancer biomarkers have highlighted the limited sensitivity and specificity of conventional serum markers for early-stage disease and emphasized that novel liquid biopsy or protein biomarkers require large-scale, multicenter validation and assay standardization before clinical implementation [33]. Thus, the present findings support the potential value of TAGLN2 as a serum candidate marker associated with gastric cancer progression, while the clinical utility of CFL1 and TAGLN2 for early T-category stratification requires further validation in larger prospective cohorts.

This research possesses specific limitations. The sample size of the prospective cohort was insufficient. Additional clinical validation of CFL1 and TAGLN2 as biomarkers in extensive, multicenter gastric cancer cohorts is essential to progress the development and clinical utilization of diagnostic test kits. The gastric cancer patients included did not receive radiation, chemotherapy, or surgical intervention. It remains uncertain whether they administered more drugs. Additional inquiry is required to ascertain whether non-anticancer medications in early gastric cancer patients influence the detection outcomes of CFL1 and TAGLN2.

## 5. Conclusions

In conclusion, our data demonstrates that CFL1 and TAGLN2 exhibit high sensitivity and specificity. These indicators are identified as innovative diagnostic biomarkers for the early detection of gastric cancer. CFL1 serum levels in gastric cancer patients were not significantly different from those in healthy individuals across seven more cancer types. No substantial difference was seen between CFL1 levels in special-condition samples from healthy persons and those from the total healthy population. This indicates a pronounced clinical specificity for CFL1. However, the matrix effect leads to different dilution factors required for the new biomarkers in actual detection. It is necessary to validate in larger multi center populations in the future and further optimize detection techniques and methods in the development of diagnostic kits.

## Figures and Tables

**Table 2:** Sample size calculation results for retrospective case-control studies.

Biomarkers	Statistical Measure	GCMN Group (ng/mL)	HC Group (ng/mL)	Theoretical Sample Size Required (Cases/Controls)	Does It Meet Statistical Power Requirements?
CFL1	Mean	4.35	2.92	106/53	Yes
	SD	3.32	3.02	-	
TAGLN2	Mean	3058.53	1825.52	18/9	Yes
	SD	1124.39	1101.43	-	

**Table 3:** Retrospective analysis of the gastric cancer diagnostic performance of CFL1 and TAGLN2.

Biomarkers	Cut Off (ng/mL)	CSE	CSP	PPV	NPV	+LR	−LR	Diagnostic Accuracy	False Negative Rate
CFL1	2.38	81.25%	57.95%	79.44%	60.71%	1.93	0.32	73.48%	18.75%
TAGLN2	2140.00	79.55%	72.73%	85.37%	64.00%	2.91	0.28	77.27%	20.45%

**Table 4:** Prospective analysis of the gastric cancer screening performance of CFL1 and TAGLN2.

Biomarkers	Cut Off (ng/mL)	CSE	CSP	PPV	NPV	+LR	−LR	Diagnostic Accuracy	False Negative Rate
CFL1	3.17	87.50%	100.00%	100.00%	98.85%	-	0.125	98.84%	12.5%
TAGLN2	3065.00	87.50%	100.00%	100.00%	98.85%	-	0.125	98.84%	12.5%

**Table 5:** Diagnostic performance of traditional tumor markers for gastric cancer.

Biomarkers	Cut off (ng/mL)	CSE	CSP	PPV	NPV	+LR	−LR	Diagnostic Accuracy	False Negative Rate
AFP	7.00	1.20%	100.00%	100.00%	39.29%	-	0.95	39.72%	98.84%
CEA	3.40	43.02%	94.55%	92.50%	51.49%	7.89	0.60	63.12%	56.98%
CA199	27.00	20.93%	89.09%	75.00%	41.88%	1.92	0.89	47.52%	79.07%
CA125	35.00	9.30%	85.45%	50.00%	60.26%	0.64	1.06	39.00%	90.70%

**Table 6:** Differential analysis of novel gastric cancer biomarkers among four molecular subtypes.

Biomarkers	Group 1	Group 2	*n*_1_	*n*_2_	*p*	*p*.adj	*p*.adj.signif
CFL1	EBV	MSI	23	49	0.479	0.575	ns
	EBV	GS	23	45	0.003	0.009	**
	EBV	CIN	23	111	0.405	0.575	ns
	MSI	GS	49	45	0.004	0.009	**
	MSI	CIN	49	111	0.909	0.909	ns
	GS	CIN	45	111	0.003	0.009	**
TAGLN2	EBV	MSI	23	49	0.981	0.981	ns
	EBV	GS	23	45	0.010	0.019	*
	EBV	CIN	23	111	0.454	0.545	ns
	MSI	GS	49	45	0.002	0.011	*
	MSI	CIN	49	111	0.411	0.545	ns
	GS	CIN	45	111	0.009	0.019	*

ns indicates no significant difference; * indicates *p* < 0.05; ** indicates *p* < 0.01.

## Data Availability

The datasets generated during and/or analyzed during the current study are available from the corresponding author upon reasonable request. The mass spectrometry proteomics data have been deposited to the ProteomeXchange Consortium (http://proteomecentral.proteomexchange.org (accessed on 31 May 2025)) via the iProX partner repository with the dataset identifier PXD061032.

## Supplementary Materials

The following supporting information can be downloaded at: https://www.mdpi.com/article/10.3390/cancers18101598/s1, Figure S1: Retrospective analysis of the standard curve for ELISA detection of CFL1 and TAGLN2; Figure S2: Prospective analysis of the standard curve for ELISA detection of CFL1 and TAGLN2; Table S1: ROC curve performance parameters of biomarkers in retrospective analysis; Table S2: ROC curve performance parameters of biomarkers in prospective analysis; Table S3: ROC curve performance parameters of biomarker combinations in retrospective analysis.

## Abbreviations

The following abbreviations are used in this manuscript:

+LR	Positive likelihood ratio
−LR	Negative likelihood ratio
ANOVA	Analysis of variance
AFP	Alpha-fetoprotein
AUC	Area under the receiver operating characteristic curve
BRCA	Breast invasive carcinoma
CA125	Carbohydrate antigen 125
CA199	Carbohydrate antigen 19-9
CEA	Carcinoembryonic antigen
CESC	Cervical squamous cell carcinoma and endocervical adenocarcinoma
CIN	Chromosomal instability
COAD	Colon adenocarcinoma
CSE	Clinical sensitivity
CSP	Clinical specificity
CV	Coefficient of variation
DDA	Data-dependent acquisition
DIA	Data-independent acquisition
EBV	Epstein–Barr virus
EC50	Half-maximal effective concentration
ELISA	Enzyme-linked immunosorbent assay
ESCA	Esophageal carcinoma
FC	Fold change
GC	Gastric cancer
GCMN	Non-metastatic gastric cancer
GS	Genomically stable
HC	Healthy control
LIHC	Liver hepatocellular carcinoma
LP	Linear predictor
LR	Likelihood ratio
LUAD	Lung adenocarcinoma
MSI	Microsatellite instability
NPV	Negative predictive value
OD	Optical density
PPV	Positive predictive value
ROC	Receiver operating characteristic
SD	Standard deviation
STAD	Stomach adenocarcinoma
TCGA	The Cancer Genome Atlas
