# Supplementary material for: Diagnostic Value of Serum CFL1 and TAGLN2 for Non-Metastatic Gastric Cancer: A Retrospective and Prospective Real-World Study
Source: Cancers (Basel). 2026 May 14;18(10):1598. doi: 10.3390/cancers18101598 (PMC13204270; doi:10.3390/cancers18101598)
Supplement: Supplementary file 1 [file cancers-18-01598-s001.zip › cancers-4274610-supplementary.pdf]

cancers

MDPI

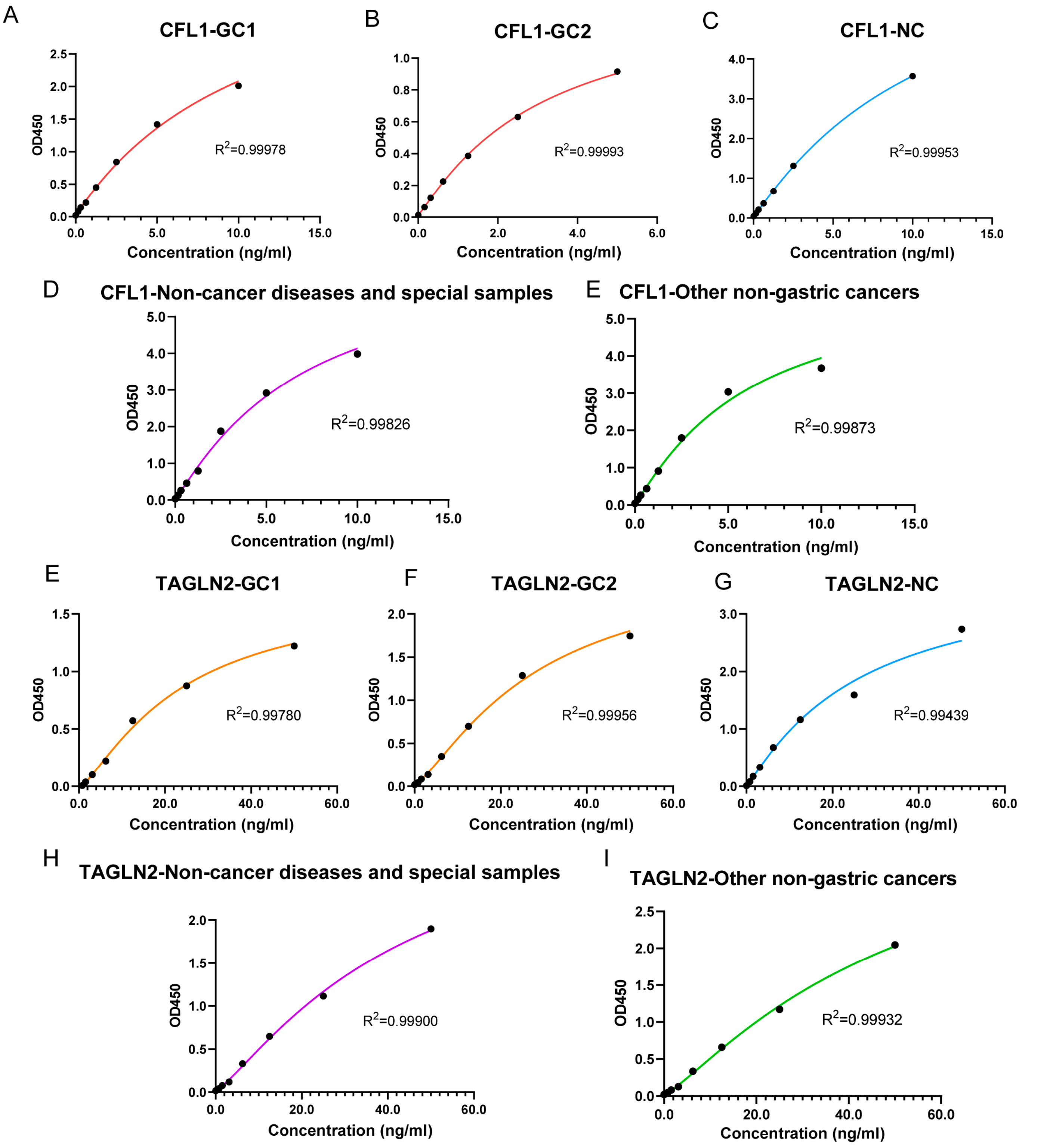

Figure S1. Retrospective analysis of the standard curve for ELISA detection of CFL1 and TAGLN2.

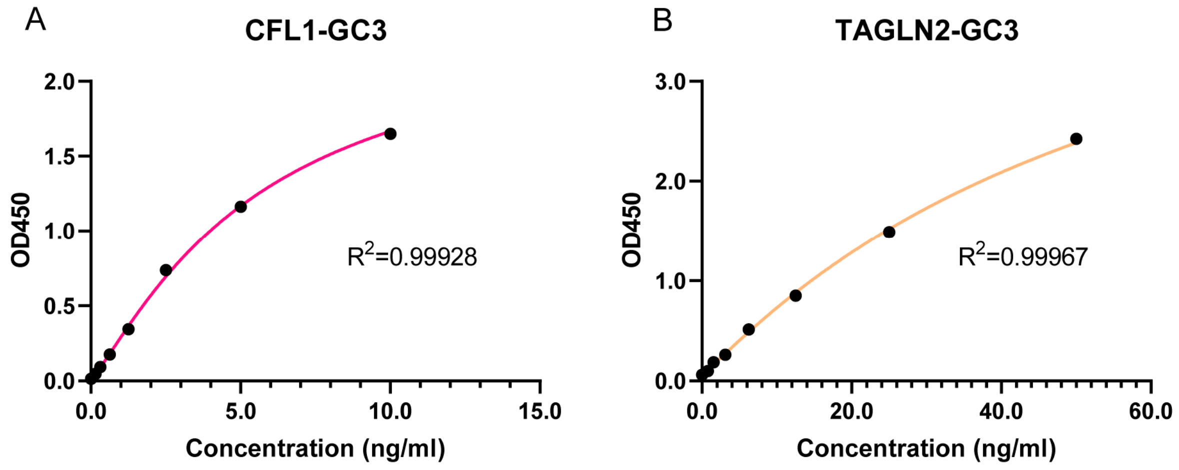

Figure S2. Prospective analysis of the standard curve for ELISA detection of CFL1 and TAGLN2.

Table S1. ROC curve performance parameters of biomarkers in retrospective analysis.

Biomarkers	Area	Std. Error	95%CI	*P*	Yoden Index
AFP	0.6481	0.04635	0.5573-0.7390	0.0031	0.2909
CEA	0.7351	0.04168	0.6534-0.8168	<0.0001	0.4446
CA199	0.5176	0.04952	0.4206-0.6147	0.7294	0.2344
CA125	0.8019	0.04016	0.7232-0.8806	<0.0001	0.4988
CFL1	0.7333	0.03448	0.6657-0.8009	<0.0001	0.3920
TAGLN2	0.8121	0.03035	0.7526-0.8716	<0.0001	0.5228

Table S2. ROC curve performance parameters of biomarkers in prospective analysis.

Biomarkers	Area	Std. Error	95%CI	*P*	Yoden Index
CFL1	0.9030	0.08539	0.7357-1.000	0.0002	0.8365
TAGLN2	0.9984	0.002580	0.9934-1.000	<0.0001	1.0000

Table S3. ROC curve performance parameters of biomarker combinations in retrospective analysis.

Biomarkers	Area	Std. Error	95%CI	*P*	Yoden Index
CFL1+TAGLN2	0.8509	0.02950	0.7930-0.9087	<0.0001	0.6250
AFP+CEA+ CA199+CA125	0.8552	0.03218	0.7922-0.9183	<0.0001	0.6416
CFL1+TAGLN2 +AFP+CEA+ CA199+CA125	0.9453	0.01832	0.9094-0.9812	<0.0001	0.7717
